# The use of common bean (*Phaseolus vulgaris*) traditional varieties and their mixtures with commercial varieties to manage bean fly (*Ophiomyia spp*.) infestations in Uganda

**DOI:** 10.1007/s10340-015-0678-7

**Published:** 2015-07-02

**Authors:** W. Ssekandi, J. W. Mulumba, P. Colangelo, R. Nankya, C. Fadda, J. Karungi, M. Otim, P. De Santis, D. I. Jarvis

**Affiliations:** National Crops Resources Research Institute (NaCRRI), P.O. Box 7084, Kampala, Uganda; National Agricultural Research Organization (NARO), P.O. Box 40, Entebbe, Uganda; Institute for Ecosystem Study, National Research Council, Verbania-Pallanza, Italy; Bioversity International, Regional Office - Uganda, P.O. Box 24384, Kampala, Uganda; Bioversity International, Regional Office - Ethiopia, c/o ILRI, P.O. Box 5689, Addis Ababa, Ethiopia; Crop Science Department, Faculty of Agriculture, Makerere University, Kampala, Uganda; Bioversity International, Via dei Tre Denari, 472/a, 00057 Maccarese, Rome Italy

**Keywords:** Bean stem maggot, Ovipunctures, Root damage, Varietal mixtures, Generalized linear mixed model, Landrace, Genetic diversity

## Abstract

The bean fly (*Ophiomyia* spp.) is considered the most economically damaging field insect pest of common beans in Uganda. Despite the use of existing pest management approaches, reported damage has remained high. Forty-eight traditional and improved common bean varieties currently grown in farmers’ fields were evaluated for resistance against bean fly. Data on bean fly incidence, severity and root damage from bean stem maggot were collected. Generalized linear mixed model (GLMM) revealed significant resistance to bean fly in the Ugandan traditional varieties. A popular resistant traditional variety and a popular susceptible commercial variety were selected from the 48 varieties and evaluated in pure and mixed stands. The incidence of bean fly infestation on both varieties in mixtures with different arrangements (systematic random versus rows), and different proportions within each of the two arrangements, was measured and analysed using GLMMs. The proportion of resistant varieties in a mixture and the arrangement type significantly decreased bean fly damage compared to pure stands, with the highest decrease in damage registered in the systematic random mixture with at least 50 % of resistant variety. The highest reduction in root damage, obvious 21 days after planting, was found in systematic random mixtures with at least 50 % of the resistant variety. Small holder farmers in East Africa and elsewhere in the world have local preferences for growing bean varieties in genetic mixtures. These mixtures can be enhanced by the use of resistant varieties in the mixtures to reduce bean fly damage on susceptible popular varieties.

**Electronic supplementary material:**

The online version of this article (doi:10.1007/s10340-015-0678-7) contains supplementary material, which is available to authorized users.

## Key message

- Ugandan farmers maintain substantial numbers of traditional common bean varieties shown to be resistant to bean fly infestation and BSM damage.
- Farmers have local preferences for growing common bean in varietal mixtures; the mixtures, when enhanced by at least 50 % of resistant varieties in a systematic random arrangement, reduced bean fly damage on susceptible popular varieties.
- This mechanism acts from the early stages, around 21 days after planting, providing a protection up to the time of maturation.

## Introduction

The common bean, *Phaseolus vulgaris,* is a staple crop in East and Central Africa serving as a food and cash crop. It is the most important plant-based protein source for the people of Uganda, providing between 20 and 25 % of the protein of the local diet (Broughton et al. 2003). More than half (53 %) of the farmers in Uganda grow beans, with the highest production in the western part of the country (Uganda Bureau of Statistics 2010; Sibiko et al. 2013). Unfortunately, bean yields have consistently remained lower than the potential yield. For instance, productivity was estimated at 1.5 t/ha, much lower than the estimated potential yields of 2.5–3.5 t/ha (Uganda Bureau of Statistics 2010). The low productivity of common beans is attributed to factors, including but not limited to, pests and diseases, declining soil fertility, plant nutritional deficiencies and drought (Allen et al. 1989). Insect pests especially the bean fly (Diptera: Agromyzidae) also known as the bean stem maggot (BSM) threaten bean production in East and Central Africa (Greathead 1968; Abate and Ampofo 1996; Ojwang et al. 2010). Talekar and Lee (1989) reported that among the bean fly species *Ophiomyia phaseoli* and to some extent *O. spencerella* are by far the most destructive and widespread in Africa, Asia, Australia and the Pacific. The insect larva bores into the stems of young plants causing plant mortality or severe reduction in growth and yield (Talekar and Lee 1989). Damage from the BSM may result in total yield losses under severe bean fly infestation, especially under low soil fertility and drought conditions (Abate and Ampofo 1996).

Conventional management techniques are used in East Africa with variable success to control the pest. Mulching (Letournaeu 1994; Byabagambi et al. 1999) and “earthing up” (Ampofo and Massomo 1998) have been found to reduce infestation, but these practices are notably labour intensive and have had limited adoption. Crop species intercropping and ensuring optimum soil water conditions are practiced in areas with sufficient available land area and water (Karel 1991; Bandara et al. 2009). Chemical insecticides, which significantly reduce infestation (Davies 1998), can also have adverse effects on the natural enemies of the BSM, resulting in pest resurgence and multiplication (Ingram 1969). Furthermore, in Uganda, many of the chemicals used to control BSM, including aldrin, aldicarb, diazinon, endosulfan, monocrotophos, thiodicarb and carbofuran, have been banned (i.e. prohibited by law) or their use has been restricted (i.e. allowed to be used only under certain situations and to be applied by specialized applicators) or both (Sustainable Agriculture Network 2011). Unfortunately, these pesticides continue to be used by farmers in Uganda because the safer products are often too expensive or not registered for use. Substantial work has been done on genetic improvement of common bean for resistance to BSM (Ojwang et al. 2009, 2011). Resistant varieties have been identified and made accessible to farmers. However, for subsistence farmers, these breeding activities have failed to achieve major impact on their food production (Ojwang et al. 2011).

Small holder farmers in Uganda avoid losses resulting from bean fly damage by early planting, seed dressing, removal of plant remains, ridging, and varietal (intra-specific bean) mixtures, all with varying success (Letournaeu 1994; Byabagambi et al. 1999; Ampofo and Massomo 1998). In Eastern Africa and the Great lakes region, small holder farmers have local preferences for growing beans in mixtures of traditional (landraces) and modern varieties, which they understand to provide resistance to local pests and diseases, and to enhance yield stability (Trutmann et al. 1993; Mulumba et al. 2012a). These common bean variety mixtures are planted with an incredible diversity of seed colour, shape and size, and the number of components in a mixture may range from 2 to 30 types (Smithson and Lenne 1996). In Uganda, bean variety richness (number of named common bean varieties) at the farmer household level has been documented to commonly range between two and six bean varieties, selected from a pool of between 10 and 27 varieties available to the farmer at the community level (Kiwuka et al. 2012; Mulumba et al. 2012b).

The main purpose of “genetic mixtures” or mixtures of varieties of the same crop, for pest and disease management, is to slow down pest and pathogen spread. The basic principle that enables varietal mixtures to reduce the severity of disease was stated by Wolfe in 1985: “Host mixtures may restrict the spread of disease considerably relative to the mean of their components, provided the components differ in their susceptibility”. The effectiveness of a given mixture to do so depends not only on the resistance available, but also on the nature and speed of the life cycles of the pathogens or pests as well as their means of spread (Marshall 1977; Razmjou et al. 2014). The mixture technique has been successfully used and well documented in pathogen management in several crops including wheat, common bean and rice (Wolfe and Finckh 1997; Finckh et al. 2000; Finckh and Wolfe 2006; Abate et al. 2000; Garrett and Mundt 1999; Mundt and Leonard 1986; Pyndji and Trutmann 1992; Zhu et al. 2000; Bowden et al. 2001). More recently, varietal mixtures for disease management have been widely experimented with in organic agriculture (Dawson and Goldringer 2012), and used in evolutionary breeding strategies (Döring et al. 2011). Less research, however, has been reported on the use of mixtures in managing insect pests. Several lines of evidence suggest that increasing genotypic diversity in crop fields could greatly improve insect pest management and crop yield in an economically and environmentally sustainable manner (Ward and Morse 1995; Tooker and Frank 2012).

Mixture trials are used to test performance of single varieties grown in pure stands against intra-specific mixtures or sets of crop varieties with non-uniform resistance. To optimally use common bean variety diversity in Uganda, the response of the different varieties, both improved and traditional, to infestation by *O. spencerella* and *O. phaseoli*, which are the most abundant and devastating BSM species in Uganda (Greathead 1968), was investigated. After which, an examination was carried out on the effect of bean varietal mixtures on BSM infestation when resistant varieties are deployed in different proportions and spatial arrangements in the varietal mixtures over the growing period of the common bean varieties.

## Materials and methods

### Site description

The study was conducted for three consecutive cropping seasons, during 2010 and 2011, at the National Crops Resources Research Institute (NaCRRI), Namulonge in Wakiso district, Uganda. The NaCRRI is located at 0°31′N, 32°35′E in central Uganda, at an elevation of 1127 m above sea level. The area receives bimodal rainfall with an annual average precipitation of 1270 mm and with temperatures ranging from 18 to 26 °C. The rainfall is distributed between two wet seasons, one lasting from March to June and the other from September to November.

### Sample collection and experimental design

Planting materials were collected for all traditional varieties encountered during focus group discussions (FGD) and household (HH) surveys from participating farmers in the districts of Nakaseke, Bushenyi and Kabale in an earlier study (Mulumba et al. 2012b). Improved varieties were obtained from the National Crops Resources Research Institute (NaCRRI)-Namulonge. Both traditional and improved varieties were screened in the field for resistance to BSM. The results of the field screening to resistance were used, combined with other criteria (see below), to select two varieties, one resistant and one susceptible for the mixture trials.

### Assessment of the response of bean genotypes to bean fly infestation

In order to assess the resistance of bean varieties to BSM infestation, 48 varieties, both traditional and improved, were screened in the field for resistance to the BSM. Bush bean varieties were planted separately from climbing varieties but close to each other in the same field. Data were collected twice during two different sowing seasons in 2010 and in 2011. The trial was set up in an alpha lattice design and replicated thrice in each season. In Uganda, the common bean growing season follows the rainy season; thus in 2010, the trial started on 25 March and finished in June. In 2011, the trial started after the second rainy season on 12 September and finished in December. Each variety was sown in four rows of 2 m in length with a spacing of 50 × 20 cm (for climber beans) and 50 × 10 cm (for bush beans). Data collection started 14 days after planting (DAP) and was repeated every 7 days until day 49. At each sampling, 20 apparently healthy bean plants were randomly selected per plot and examined for bean fly infestation symptoms for estimation of incidence (Parker et al. 2000). Incidence was expressed as a percentage of infested plants per plot. In addition, data were collected on counts of bean fly pupae recovered from dissected dead plants.

We evaluated the incidence (as the proportion of infested versus non-infested plants) and severity (as the number of pupae observed) using a generalized linear mixed model (GLMM). This approach allows modelling the sources of variation and correlation that arise from grouped data by combining the properties of linear mixed models and generalized linear models (Bolker et al. 2009). GLMM is well suited for our dataset for which observations were collected during 2 different seasons, from 14 up to 49 DAP. GLMMs allowed us to take account of the structures of our dataset that might influence our inferences. Incidence was transformed in a binomial descriptor (presence/absence), while for the number of pupae, after a visual inspection of the frequencies distribution, a Poisson distribution of the error was used (Table 1). The name of varieties was used as the fixed factor, while the DAP nested in the season and blocks with bush or climber beans were used as random effects. The significance of the two GLMMs was tested versus a null model (no incidence and severity differences among varieties) using a likelihood ratio test (LRT).

**Table 1:** General linear mixed models (GLMMs) used in order to (1) analyse tolerance of different bean genotypes to BSM (2 models); (2) analyse the incidence of bean fly on the susceptible and resistant varieties separately controlling, in turn, for placement and proportions (2 models for each variety); (3) investigate the effect of different types of mixture (different combination of arrangements and proportions); (4) the dynamic of the incidence of root damage across different mixture types over time for the susceptible variety

Focus	Dependent variable	Fixed effect	Random effect	Family
Genotype tolerance	Incidence	Varieties name	Season/DAP, bean type	Binomial
Genotype tolerance	No of pupae	Varieties name	Season/DAP, bean type	Poisson
Nabe 4 (*susceptible variety*)	Incidence	Placement	Season/DAP, proportion	Binomial
	Incidence	Proportion	Season/DAP, placement	Binomial
Kasirira (*resistant variety*)	Incidence	Placement	Season/DAP, proportion	Binomial
	Incidence	Proportion	Season/DAP, placement	Binomial
Mixture	Root damage	Proportion * Placement	Season/DAP	Poisson
Susceptible variety	Root damage	Mixture combinations * DAP	Season	Poisson

For each GLMM is reported: the focus of the analysis, the dependant variable, the fixed effects and the random effect formula, and the family of the error distribution. Asterisks indicate that the interaction between two fixed effects was also considered in the models

### Relationship between BSM damage and yield reductions

The 48 bean genotypes were also screened for yield over the two seasons. Yield data were taken at physiological maturity when whole plots were harvested, threshed, dried, and the seed yield recorded. In addition, the number of dead plants per plot was counted and compared to the total number of emergent plant at DAP 14. For both the two seasons, the relationship between yield loss and percentage of dead plants was tested by a GLM. Significance was obtained by using a *F* test.

### Assessment of the effect of varietal mixtures on bean fly infestation and damage

In order to assess the effect of varietal mixtures on infestation and damage by bean fly on common beans, two bush bean varieties (one susceptible and one resistant) were used. The selection of the two varieties used in the variety mixture was based on three criteria: (1) their response to pest infestation and damage according to the results of the assessment of genotypes reaction to BSM; (2) the popularity of the varieties amongst the farmers in the communities; and (3) the ease to visually distinguish the varieties in the field at all growth stages.

The mixture trials were conducted during the 2011 cropping season. A first sowing was done at the on-set of rains, whilst a second was sown 3 weeks later in a separate field. A total of six treatments were laid out consisting of two spatial arrangements: Alternate-row versus systematic random mixture arrangements; each with three mixture proportions: 25:75, 50:50 and 75:25 of susceptible to resistant varieties, respectively (see Appendix A in the Supplementary Materials for a schematic diagram of arrangements). Two controls of pure stands one with a resistant variety and the other with a susceptible one were included. Treatments were laid out in a randomized complete block design with three replicates.

Each plot was surrounded on all four sides by a 2-m guard area of tilled ground to minimize inter-plot interference. The varieties were planted in plots consisting of 12 5-m-long rows with 50 × 10 cm spacing between rows and plants, respectively, and left under natural bean fly infestation. The resistant and susceptible varieties were identified in a row by their appearance. The resistant variety had small leaves, purple stems, petioles and flowers, while the susceptible variety had broad leaves, green stems and petioles and pink flowers. Data were collected weekly starting at 14 DAP and ended at 49 DAP. Destructive sampling was done on the same day on all treatments. At each sampling, 20 plants, 10 of each of the tolerant and susceptible variety, were randomly sampled from middle rows in each plot and examined for the presence of larvae and/or pupae by dissecting their stems in turn. Incidence of bean fly was recorded as either 0 or 1 for the absence or presence of larvae and/or pupae. Severity of bean fly infestation was expressed as the number of pupae on each of the sampled plants. Root damage was scored using a visual root damage scale of 1–5, where 1 = no damage, 2 = slight damage, 3 = moderate damage, 4 = severe damage and 5 = complete damage and plant death.

In order to evaluate how the arrangement type (systematic random versus alternate-row mixtures) and the proportion of resistant and susceptible varieties affect the incidence of bean fly on the mixture trials, we used different GLMMs (Table 1). Initially, GLMMs were built in order to evaluate the effect of placement and proportion of plants in the mixture on the incidence. Because our aim was to understand the effect of each of these effects on the resistant and susceptible component of our mixture, we designed four different models in order to analyse the incidence of bean fly on the susceptible and resistant varieties separately controlling, in turn, for placement and proportions (Table 1). Susceptible and resistant pure stands were included in the GLMMs as control. In all four GLMMs, DAP nested in the season was included as random effect. All the four GLMMs were tested against a null model by LRT.

In order to fully investigate the effect of different types of mixture (different combination of arrangements and proportions) on the root damage level, a fifth model was built using placements, proportions and their interactions as fixed effects (Table 1). Pure stands were dropped from this analysis, and the DAP nested in the seasons was included as the random effect. The effect of all the possible combinations of the two fixed factors and a null model were estimated. The best fit of the obtained models was evaluated using the Akaike Information Criteria (AIC). Finally, to evaluate the dynamics of the incidence of BSM over time on the susceptible variety, we performed a further GLMM using root damage as the dependent variable, the mixture combinations (a new variable with 7 levels created by the 6 combinations of different proportions and placements, plus the pure stand) DAP and their interaction as the fixed effects, and planting season as the random effect (Table 1). The fit of the full model versus the reduced models and null model was tested using AIC. All the GLMMs were obtained using the R package lme4 version 1.1.7 (Bates et al. 2014). Graphs were obtained using the R package “effects” version 3.0.1 (Fox 2003; Fox and Hong 2009).

## Results

### Response of bean genotypes to bean fly infestation and bean stem maggot (BSM) damage

**Fig. 1:**
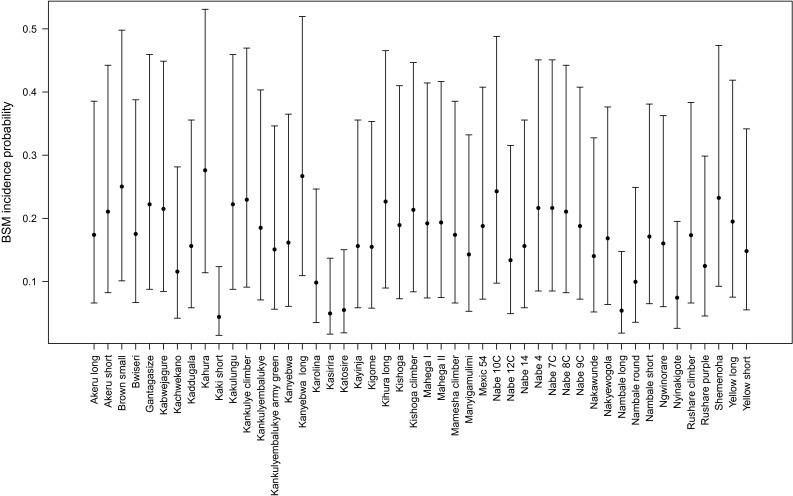
Estimated infestation incidence (*points*) and 95 % confidence interval (*bars*) for the 48 screened genotypes. *Nambale long, Kasirira, Katosire* and *Kaki short* show a low incidence of BSM. Other four varieties, *Kahura, Kanyebwa long, Nabe 10C* and *Shemenoha*, show the highest incidence of BSM

**Fig. 2:**
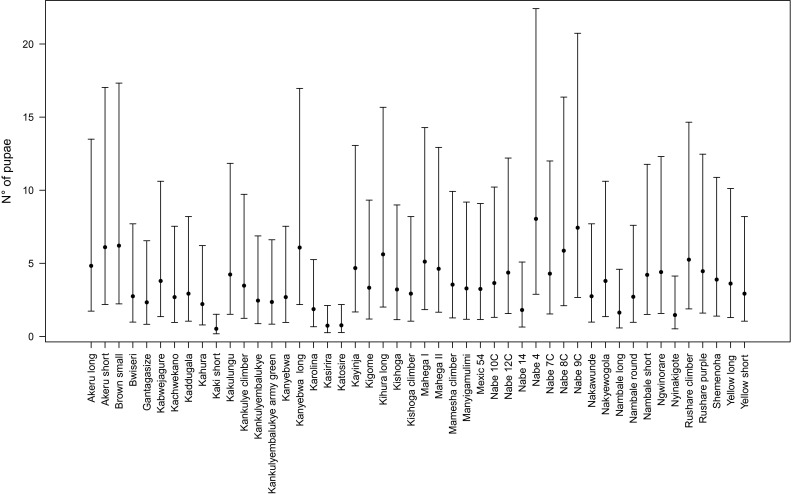
Estimated infestation severity (*points*) and 95 % confidence interval (*bars*) for the 48 screened genotypes. *Nambale long, Kasirira, Katosire* and *Kaki short* show low infestation severity, while the two modern varieties, *Nabe 4* and *Nabe* 9C, show the highest severity

The LRT rejected the null hypothesis of no differences of BSM incidence (*P* < 0.001) and severity (*P* < 0.001) among varieties. According to the estimated incidence values (Fig. 1), four varieties, *Nambale long, Kasirira, Katosire* and *Kaki short*, show a particularly low incidence of BSM. Four other varieties, *Kahura, Kanyebwa long, Nabe 10C* and *Shemenoha*, show the highest incidence of BSM. According to severity estimates (Fig. 2), the four more resistant varieties are also the four varieties showing lower severity. The two modern varieties, *Nabe 4* and *Nabe 9C*, showed higher severity (Fig. 2).

### Relationship between BSM damage and yield reduction

**Fig. 3:**
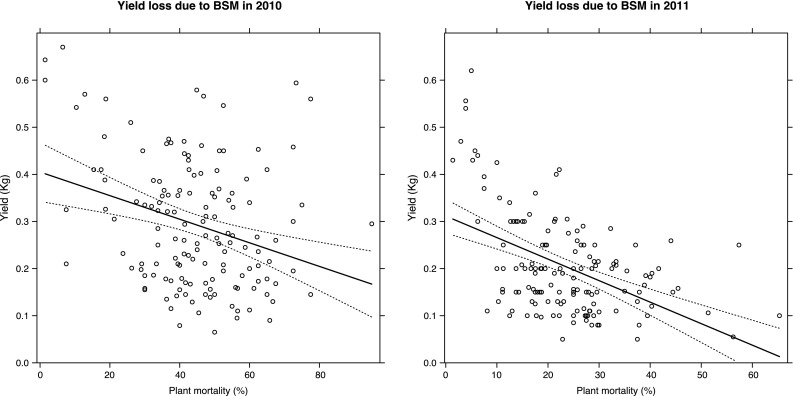
Yield loss explained by the observed percentage of dead plants in each plot. *Solid lines* represent fitted values, while *dotted lines* represent 95 % confidence interval

A significant relationship was found between the percentage of dead plants and the yield loss. Despite the different percentage of dead plants recorded in the 2 years, both in 2010 (*F* = 14.087, *P* < 0.001) and in 2011 (*F* = 42.477, *P* < 0.001), the GLM highlights a significant decrease in yield with the increase in damage (Fig. 3).

### Effect of varietal mixtures on bean fly infestation and BSM damage

Based on the results of the resistance screening of bean genotypes to bean fly, and considering the following other selection criteria, *Kasirira* (the resistant traditional variety) and *Nabe 4* (the commercial susceptible variety) combination were selected for the mixture trial. Both varieties are known to be very popular from earlier FGD and household surveys, described in Mulumba et al. 2012a, b. Furthermore, the two varieties could easily be distinguished by their morphological traits (e.g. leaves, colour of stem, petiole, flower and pods). Both varieties are bush beans. Bush beans were selected rather than climbing beans as the majority of farmers in Uganda grow bush beans. *Kasirira* was selected over the other resistant varieties because of its popularity with Ugandan farmers, i.e. the variety is grown by many farmers compared to other bean varieties especially in the eastern and northern parts of Uganda. *Nabe* 4 was selected as the susceptible variety, as this variety is very popular and marketable throughout the country compared to the other susceptible choices.

**Fig. 4:**
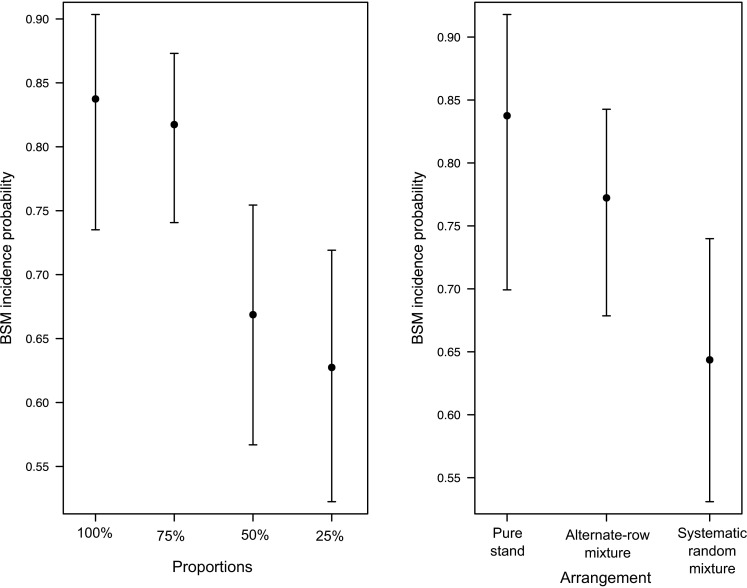
Estimated BSM infestation probability (*points*) with 95 % confidence interval (*bars*) for the susceptible *Nabe* 4 variety according to different proportions in the mixture (on the *left*) and different arrangement type (on the *right*). In both cases, proportions and arrangement types were compared to the incidence probability of the *Nabe* 4 pure stand

The GLMM analysis showed that the mixture arrangement type (alternate-row and systematic random mixtures) has a significant impact on reducing BSM in *Nabe 4* (the susceptible variety), resulting in a decrease of the probability of incidence of BSM (LRT *P* < 0.001, Fig. 4). However, the pest incidence in alternative-row mixtures was not significantly different from that in pure susceptible stands (*P* = 0.7, Table 2).

**Table 2:** Estimated values of incidence for *Nabe 4* according to the arrangement type

Fixed effects	Estimated	Std. error	*Z* value	*P*
Pure stand (intercept)	1.63	0.40	4.064	<0.001
Alternate-row mixture	−0.42	0.42	−0.921	0.36
Systematic random mixture	−1.04	0.45	−2.317	<0.001

The alternate-row mixture arrangement did not show a significant decrease compared to the pure stand. Standard error of estimated values and *z*-test are reported

When we tested the effect of the proportion of susceptible and resistant plants in the mixture, we were able to reject the null model of no effect of different proportion of varieties in the mixtures (LRT *P* < 0.001). There was a significant reduction of BSM incidence with respect to the pure stand when *Nabe 4* is represented by 25 % (*P* = 0.002) or 50 % (*P* = 0.01) of the plants in the mixture. In contrast, when the susceptible variety *Nabe 4* represents 75 % of the plants in the mixtures, the incidence of BSM is not significantly different (*P* = 0.7) compared to the pure stand susceptible control (Table 3). The two GLMMs were repeated for the resistant variety (*Kasirira*) alone, but the null model could not be rejected in both the cases, i.e. neither arrangement type nor different proportion of *Nabe 4* in the mixture caused a reduction of resistance of *Kasirira* to BSM.

**Table 3:** Estimated values of incidence for *Nabe* *4* according to the proportion of susceptible (S) and resistant (R) varieties in the mixture

Fixed effects	Estimated	Std. error	*Z* value	*P*
Pure stand (intercept)	1.63	0.31	5.250	<0.001
25R:75S	−0.14	0.37	−0.380	0.7
50R:50S	−0.93	0.36	−2.570	0.01
75R:25S	−1.11	0.36	−3.071	0.002

A significant decrease of incidence can be observed when at least 50 % of the *Kasirira* resistant variety is present in the mixture. The standard error and *z*-test are reported

**Fig. 5:**
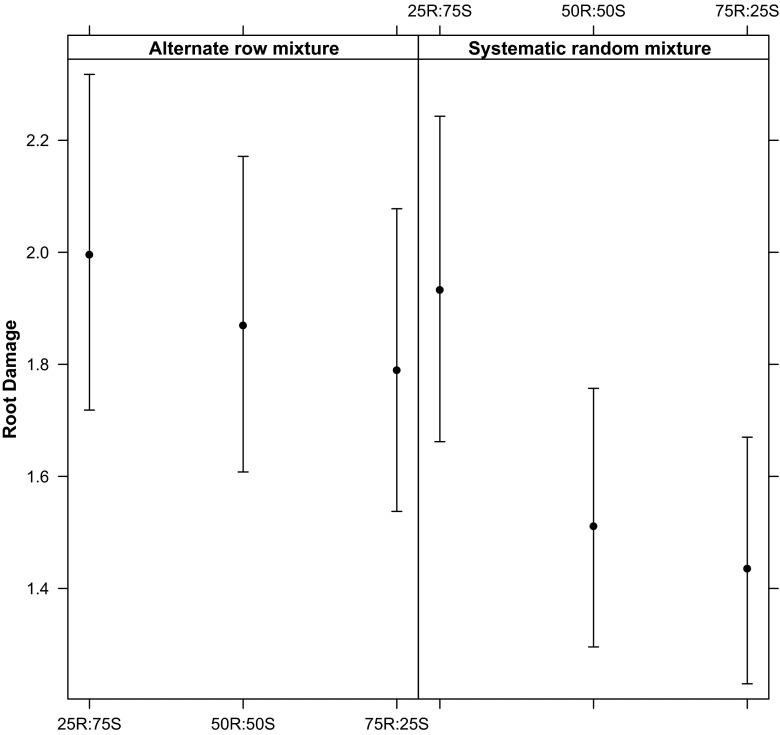
Estimated root damage (*points*) and 95 % confidence interval (*bars*) in different mixture combinations. Random mixtures, with at least 50 % of the resistant *Kasirira* variety, show the highest reduction of root damage

When the effect of the proportion and arrangement type (excluding the 2 pure stand controls) was analysed, the full model, including the interaction of the two effects, was found to be the best model according to the AIC (Table 4). The resistance level of the mixture increased with the increase of resistant *Kasirira* variety proportion in the mixture (Fig. 5). However, it is evident that with the same proportions in the mixtures, the systematic random arrangement causes a higher reduction of BSM incidence than the alternate-row arrangement (Fig. 5).

**Table 4:** Four GLMMs were tested against a null model of no effect of proportion and arrangement type in the mixtures

Model	df	AIC
Proportion * arrangement	8	13042.70
Proportion + arrangement	6	13053.09
Proportion	6	13095.75
Arrangement	6	13091.90
Null model	3	13146.82

The full model shows the best AIC score. The asterisk indicates that the interaction between fixed effects was also tested (full model)

### Root damage over time

**Fig. 6:**
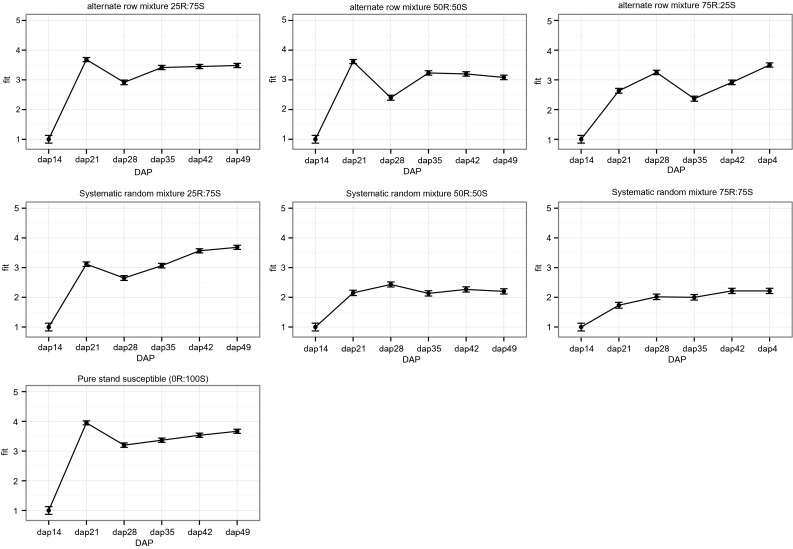
Estimated root damage with standard error for the susceptible *Nabe 4* variety from DAP 14 up to DAP 49 in six different mixture types compared to the susceptible pure stand (100 % *Nabe* 4). A steep increase in root damage is observed for alternative-row mixtures, while random mixtures with at least 50 % of the resistant *Kasirira* variety remained at lower damage levels during the entire maturation time

The GLMM analysis of root damage of the susceptible variety *Nabe4* was best explained by the models that take into account the mixture type, the maturation stage and their interactions (Table 5). However, the model that did not consider the interaction term of the two factors showed only a slightly lower AIC (Table 5). From Fig. 6, it is evident that the significant reduction of the root damage occurs in the systematic random mixtures with at least 50 % of the resistant *Kasirira* variety in the mixture. The increased effect occurs at 21 days after planting (DAP); the time period when in the other mixtures we recorded the highest level of root damage. For alternate-row mixtures, at least 75 % of the resistant *Kasirira* variety was needed to have an effect at the 21 DAP. The protection to BSM-produced damage is then prolonged up to DAP 49.

**Table 5:** GLMM was used to investigate the effect of mixture type and maturation stage on the susceptible variety

Model	df	AIC
Mixture type * DAP	43	8161.117
Mixture type + DAP	13	8161.947
DAP	8	8791.698
Mixture type	7	8348.701
Null model	2	8978.452

AIC suggests that the model considering both the factors and their interaction is the best. The asterisk indicates that the interaction between fixed effects was also tested (full model)

## Discussion

### Differential susceptibility of common bean varieties

Most, if not all, known resistance to arthropod pests used in breeding programmes is derived from varieties collected from farmers who traditionally grow them in genetically diverse systems (Brown 1999). Materials to improve common bean for resistance to BSM in eastern and southern Africa have come mostly from screening traditional varieties predominantly from gene bank accessions (Abate et al. 2000; Ojwang et al. 2009, 2010). Ojwang et al. (2010) screened 64 bean genotypes and identified seven resistant traditional bean varieties. Similarly, Ogecha et al. (2000) identified 13 out of 66 screened traditional varieties to be tolerant to BSM. The bean varieties managed by small holder farmers in Uganda evaluated in this study showed significant variation in respect to BSM infestation and damage. Many of the traditional common bean varieties that Ugandan farmers continue to grow in their fields are resistant to bean fly infestation and BSM damage. Several morphological features have been associated with BSM resistance in some crops. For example, Chiang and Norris (1983) noted that leaf area, trichome density of the under surface of leaves, stem diameter and moisture content of stems influenced BSM infestation of soybean. This was confirmed by Dharmasena and Fernando (1988) working on cowpeas who also showed that varieties with smaller leaf areas, small stem diameter, and lower stem moisture content manifested greater resistance to BSM attack. Such characteristics may in part have led to the observed differential response of the Ugandan varieties to BSM infestation and damage. The varieties which were least affected by BSM, namely *Kasirira*, *Katosire* and *Kaki short,* indeed have small seeds, small leaf areas and small stems compared to varieties such as *Kanyebwa*, *Nabe 4*, *Nakyewogola* with much bigger leaves and stems.

### Resistance and yields through mixtures

Both the spatial arrangement and the proportion of components in the mixtures influenced the incidence of BSM and root damage, with the highest decrease in damage registered in the systematic random mixture with at least 50 % of resistant variety. Despite different percentage of mortality observed between the 2010 and 2011 cropping seasons, a negative relationship was consistently found between yield and the number of dead plants. This indicates that the use of these bean mixtures is a promising approach to help farmers increase their yield stability, as the mixtures improve resilience of the farmers’ production system by protecting susceptible varieties from fluctuations in pest infestation.

Pest populations will spread rapidly from one plant to another once the pests invade the field if all the plants in the field are susceptible to the same pest species (Tooker and Frank 2012). Plants of the resistant variety enhance spatial isolation (distance between susceptible plants) and may act as physical barriers that reduce the numbers and activity of vectors or pests (Sserubombwe et al. 2001). In a systematic random mixture arrangement, such isolation is more pronounced compared to the alternate-row arrangement. Increasing the proportion of resistant plants in mixture plots further increases the isolation of the preferred (susceptible) host plants hence making it harder for the bean flies to locate susceptible hosts. In contrast, there are no spatial discontinuities in monocultures of a susceptible variety. The results of this study are in agreement with other work on varietal mixtures that suggest that the further susceptible plants are isolated from each other, the less the chances that pests, vectors and pathogens will move between them (Wolfe 1985; Tooker and Frank 2012). The barrier effect of mixing different entities to restrict BSM movement has also been recorded when intercropping bean with other crops, showing a reduction in the number of larvae and pupae and the death of bean plants (Karel 1991; Peter et al. 2009; Bandara et al. 2009). Interestingly, Peter et al. (2009) also noted the importance of ensuring sufficient proportions within their inter-cropped populations of at least one-third bean plants with two-thirds maize plants to have the best relative yield advantage for the management of bean stem maggots.

Harvesting varieties separately is an acceptable practice for Ugandan farmers. The improved variety *Nabe4* starts to ripen approximately a week before *Kasirira* (the traditional resistant variety). Therefore, even if grown in mixtures, the varieties can be harvested separately owing to their differential maturation periods. For small holder farmers in Uganda, with limited land area for crop production, the choice of allocating space to inter-crop bush beans with other crops, or to plant common bean mixtures, or to do both, as a pest management strategy, will clearly also depend on each individual household’s criteria and choices to meet production needs and cultural or dietary preferences.

### Dynamics of infestation

Root damage in the young bean plants was evident very early in our study, only 21 days after planting. Normally, the bean fly larval (maggot) stage lasts 7–10 days, and the pupal stage 9–10 days, resulting in a life cycle of approximately 3 weeks, although variation in this life cycle has been observed to extend for the larval stage up to 22 days, and the pupal stage up to 20 days (Waterhouse 1998). A linear or exponential growth of root damage over the time for mixtures (both with alternate-row or systematic random arrangements) was not observed with less than 50 % of the traditional resistant variety. In these cases, the root damage reached a plateau early and remained constantly high after day 21. In contrast, in the systematic random mixture with at least 50 % of the resistant variety, a significant reduction of infestation since the early growth stages was observed. This “protection effect” is prolonged up to maturation, suggesting that the early stages of the common bean growing period are the more critical to manage and reduce BSM infestation. The enhanced resistance of systematic random mixtures might be explained by the fact that the presence of the resistant variety can provide a physical and/or chemical barrier to the spread of bean fly on the susceptible variety together with a reduction in the likelihood of the pest to recognize a susceptible host. The BSM life cycle and the duration of availability of young tender leaves (Peter et al. 2009), together with possible differences in nutritional value of the host affecting the rate of development and population dynamics of insect pests (Razmjou et al. 2014), may be reasons for the peak of pupae and root damage at 21 days for susceptible varieties when not in varietal mixtures (Fig. 4). This indicates that the effect of the mixture may therefore be strongest in the first 20 days of the bean plant life cycle. The mechanism acts from the early stages (21 DAP), providing a protection to the bean plants up to the time of maturation. The implication is that a genetic mixture with a systematic random spatial arrangement, containing at least 50 % resistant plants in the mixture, is an effective method to manage BSM early in the season.

## Conclusion

Ugandan farmers continue to maintain traditional common bean varieties that are resistant to bean fly infestation and BSM damage in their agricultural production systems. This diversity has a high potential to have impact if integrated in both conventional and participatory plant breeding programmes. Pest susceptibility often joins a complex list of criteria that determine the choice of these farmers on what variety or group of varieties to plant to meet their production needs. Small holder farmers in East Africa have local preferences for growing bean varieties in genetic mixtures. This study has provided further evidence that such genetic mixtures provide an affective buffering effect to pest damage and a potential yield advantage. Inclusion of resistant varieties in the mixtures can reduce BSM damage on susceptible popular varieties, particularly when mixtures are planted in a systematic random arrangement with the resistant genotype constituting at least 50 % of the mixture. This mechanism acts from the early stages providing a protection up to maturation for the bean plants.

## Author contribution statement

SW carried out the research and drafted the manuscript; DIJ, PDS, JWM and CF contributed to the research design and supervised the experiments; PC analysed the data; RN supervised the experiments and edited the manuscript; JK and MO supervised the research; DIJ and JWM conceived and designed the research; PC, RN and DIJ edited the manuscript. All the authors read and approved the manuscript.

## Electronic supplementary material

Supplementary material 1 (PDF 1158 kb)

Appendix A: Design of the experiment used to assess the effect of varietal mixtures on bean fly infestation and BSM damage.
