# Supplementary material for: The use of common bean (Phaseolus vulgaris) traditional varieties and their mixtures with commercial varieties to manage bean fly (Ophiomyia spp.) infestations in Uganda
Source: J Pest Sci (2004). 2015 Jul 2;89:45–57. doi: 10.1007/s10340-015-0678-7 (PMC4757615; doi:10.1007/s10340-015-0678-7)
Supplement: Supplementary file 1 — Supplementary material 1 (PDF 1158 kb) [file 10340_2015_678_MOESM1_ESM.pdf]

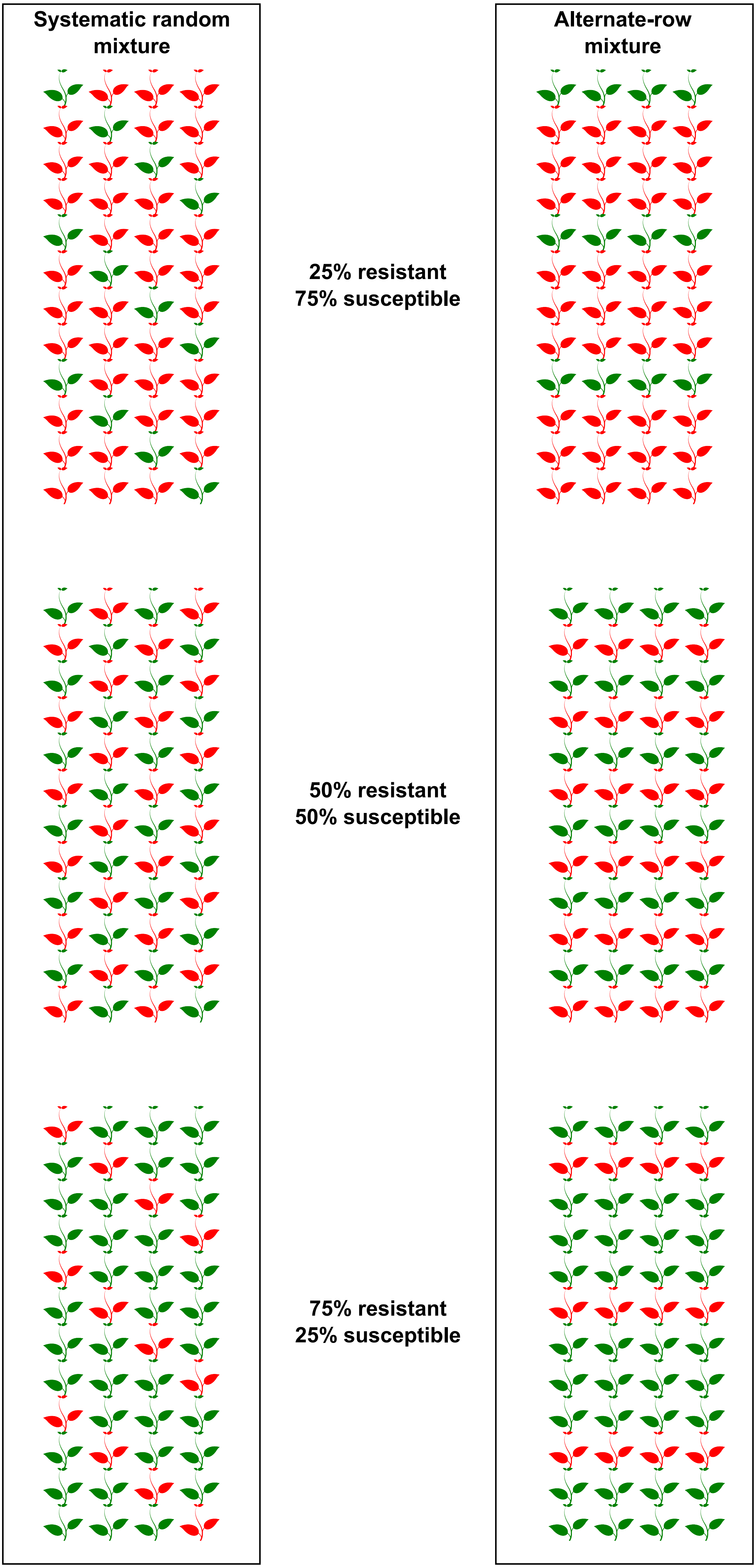
Systematic random mixture
Alternate-row mixture
25% resistant
75% susceptible
50% resistant
50% susceptible
75% resistant
25% susceptible
